# Drastic change of magnetic anisotropy in Fe_3_GeTe_2_ and Fe_4_GeTe_2_ monolayers under electric field studied by density functional theory

**DOI:** 10.1038/s41598-021-96639-3

**Published:** 2021-09-02

**Authors:** Dongwook Kim, Changhoon Lee, Bo Gyu Jang, Kyoo Kim, Ji Hoon Shim

**Affiliations:** 1 Department of Chemistry, Pohang University of Science and Technology (POSTECH), Pohang, 37673 Korea; 2 Max Planck POSTECH Center for Complex Phase of Materials, Pohang University of Science and Technology, Pohang, 37673 Korea; 3 Korea Institute for Advanced Study (KIAS), Seoul, 02455 Korea; 4 Korea Atomic Energy Research Institute (KAERI), Daejeon, 37673 Korea; 5 Department of Physics, Pohang University of Science and Technology (POSTECH), Pohang, 37673 Korea; 6 Division of Advanced Materials Science, Pohang University of Science and Technology (POSTECH), Pohang, 37673 Korea

**Keywords:** Ferromagnetism, Electronic structure

## Abstract

Magnetic anisotropy energy (MAE) is one of the most important properties in two-dimensional magnetism since the magnetization in two dimension is vulnerable to the spin rotational fluctuations. Using density functional theory calculation, we show that perpendicular electric field dramatically enhances the in-plane and out-of-plane magnetic anisotropies in Fe_3_GeTe_2_ and Fe_4_GeTe_2_ monolayers, respectively, allowing the change of easy axis in both systems. The changes of the MAE under the electric field are understood as the result of charge redistribution inside the layer, which is available due to the three-dimensional (3D) network of Fe atoms in the monolayers. As a result, we suggest that due to the unique structure of Fe_n_GeTe_2_ compounds composed by peculiar 3D networks of metal atoms, the MAE can be dramatically changed by the external perpendicular electric field.

## Introduction

Magnetic van der Waals (vdW) materials are in interest for recent years, mainly as a platform for exfoliatable two-dimensional magnetic systems. Many materials have been reported with various types of magnetisms^1–26^ and some were experimentally verified to have magnetisms even in the monolayers^1–5^. *M*P*X*_3_ (*M* = Metal, *X* = Chalcogen) family took the most interest at the beginning of the studies on vdW magnetism, since various combinations of *M* and *X* were already reported and studied^6–11^. Soon after, CrI_3_ and Cr_2_Ge_2_Te_6_ have been studied since they were ferromagnetic upon monolayer or bilayer limits^1, 2^. Recently, Fe_3_GeTe_2_ is under the most interests since it is a ferromagnetic metal showing Curie temperature of 220 K at bulk, which is a very high value among the reported vdW materials^5, 12–24^. Moreover, it is reported that Fe_3_GeTe_2_ shows ferromagnetism even in the limit of monolayer^5^. Recently, Fe_4_GeTe_2_^25^ and Fe_5_GeTe_2_^26^, which have structure similar to Fe_3_GeTe_2_, were synthesized and showed ferromagnetism up to 280 K and 310 K, respectively. Because of the similarity to Fe_3_GeTe_2_, they are expected to be ferromagnetic at the monolayer limit.

Compared to other vdW materials, Fe_*n*_GeTe_2_ (*n* = 3, 4, 5) (FGT) have a very rare feature, intra-layer three-dimensional (3D) network of the metal ions sealed by Te ligand sheet^25^. The intra-layer 3D network allows a direct metal–metal bonds along both planar and perpendicular directions. The increased number of neighboring magnetic ions is believed to be the origin of strong itinerant ferromagnetism with high Curie temperature^25^. However, the Curie temperature in the monolayer is still expected to be lower than that in bulk since the magnetic ordering becomes more sensitive to the thermal fluctuation in two-dimensional (2D) system. Indeed, it is experimentally confirmed that the Curie temperature of Fe_3_GeTe_2_ is much lower in the monolayer than the bulk^20^. Since the magnetic anisotropy energy (MAE) is usually much smaller than the exchange energy, the spin rotational fluctuations are the key ingredients that weakens the magnetic ordering in 2D system, as it is known that the gapless Goldstone mode disables long-range ordering in 2D system according to Mermin–Wagner theorem if the MAE becomes zero^27–29^. In real materials, however, the MAE allows the magnetic ordering by restricting the rotational fluctuation modes. The uniaxial MAE increases the Curie temperature rapidly, while the planar magnetic anisotropy disables magnetic ordering since the spin O(2) symmetry gives the Goldstone mode from the free planar rotation. Thus, the easiest way to tune the magnetism of monolayer magnetic system is to control the MAE. There have been studies on the MAE change of Fe_3_GeTe_2_, with the strain effect on the monolayer^21^ or the hole doping on the bulk system^22^.

**Figure 1:**
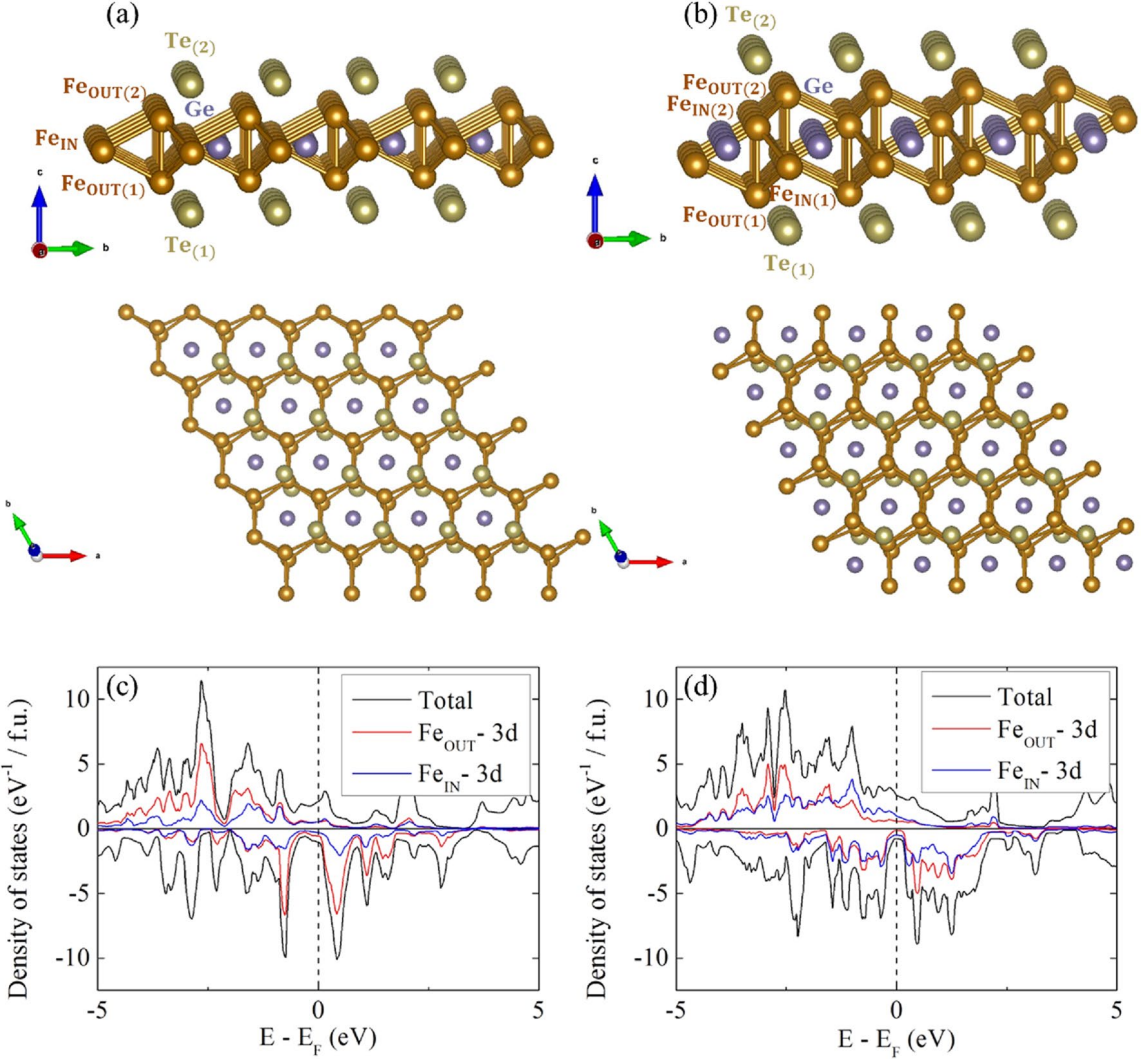
Crystal structures of (**a**) Fe_3_GeTe_2_ and (**b**) Fe_4_GeTe_2_ monolayers. Space group numbers are 187 and 164 for Fe_3_GeTe_2_ and Fe_4_GeTe_2_ respectively without electric field, but as electric field is induced, both changes to 156. Both compounds have 3D networks of Fe atoms embracing Ge atoms at the center of the network and sealed by Te atoms. The total and Fe partial DOSs of (**c**) Fe_3_GeTe_2_ and (**d**) Fe_4_GeTe_2_ monolayers, showing that the states near the Fermi level are dominated by Fe atoms.

In this work, by using first principles calculations, the MAE changes by the perpendicular electric fields in the Fe_3_GeTe_2_ and Fe_4_GeTe_2_ monolayers are investigated. Since there are 3D networks of Fe atoms in FGT monolayers as shown in Fig. 1a,b, the electric field can move the electrons from one side of the layer to the other side. Thus, the electric field effect can be schematically understood as doping oppositely on Fe atoms in each sides of the layer. As a single unit of the FGT monolayer, the MAE is drastically changed by the external electric field, so we suggest that this system can be applied to the efficient *in-situ* magnetism control in spintronic devices.

## Methods

For this study, density functional theory based on projector augmented wave (PAW) method implemented in Vienna ab initio simulation package (VASP) was used for entire work^30–33^. For the exchange correlation potential, the generalized gradient approximation (GGA) by Perdew-Burke-Ernzerhof (PBE) was used^34^. The cut-off energy for plane wave basis set of 700 eV was used. For *k*-mesh, $21\times 21\times 1$ centered at $\Gamma$ point was used. Crystal structures were obtained by the structure relaxation with the same options described above. The MAE, defined as *E*(001)—*E*(100) were determined by the comparison between the total energy of self-consistently calculated results with spin direction along *z* (001) and *x* (100) axis.

## Results and discussions

### Intralayer electron redistribution by the electric field

Crystal structures of Fe_3_GeTe_2_ and Fe_4_GeTe_2_ monolayers are shown in Fig. 1a,b, respectively. Both of these materials have the 3D networks of Fe atoms allowing stable itinerant ferromagnetism. Moreover, the 3D networks allow the electrostatic potential bias between Fe atoms by the perpendicular electric field. Since these systems are metallic, the continuous change of the induced electric field can result in charge transfer from one side to another side of the monolayer.

Densities of states (DOS) near the Fermi level are dominated by Fe-*d* orbitals, as shown in Fig. 1c,d for Fe_3_GeTe_2_ and Fe_4_GeTe_2_, respectively. Thus, the charge redistribution by the external electric field or carrier doping are expected to influence mostly on Fe-*d* orbital occupation. However, owing to the Fe-Te hybridizations, the charge redistribution by the electric field can indirectly influence on Te atoms which provide strong spin–orbit coupling. Thus MAE can be changed by the charge redistribution.

**Figure 2:**
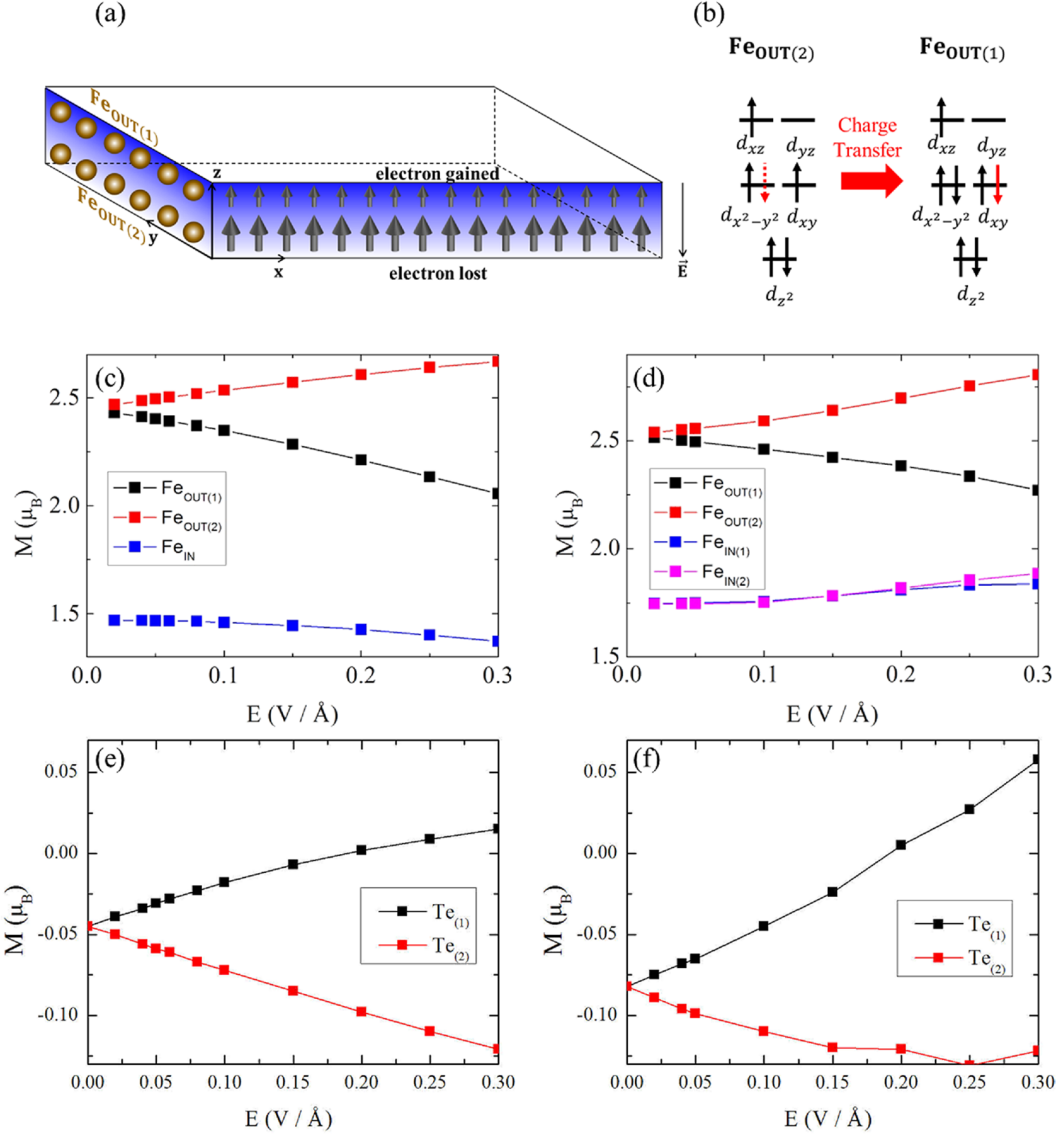
(**a**) Schematic figure of electric field effect on monolayer FGTs on the spin moments of each side of the layer, based on (**b**) the analysis on the relation between net spin moment and the number of electrons for crystal field splitting in case of outer Fe atoms in *d*^6^ occupation. (**c**,**d**) shows the electric field dependence of the magnetic moments of Fe atoms in Fe_3_GeTe_2_ and Fe_4_GeTe_2_ respectively, which agree to the expectation in (**b**). (**e**,**f**) shows the electric field dependence of the magnetic moments of Te atoms in Fe_3_GeTe_2_ and Fe_4_GeTe_2_ respectively, showing the strong antiferromagnetic interaction and hybridizations between Te atoms and their adjacent Fe atoms.

As the perpendicular electric field is induced, the electrostatic potential biases enter in these systems and move the electrons from one side of the layer to the other, as schematically shown in Fig. 2a. Since these systems have *z*-inversion symmetry in the structures, we can consider the charge redistribution as charge transfers between *z*-inversion paired atoms. Among the Fe atoms, Fe_OUT_, which are located far from the center, get large charge redistribution by the electric field due to the large electrostatic potential bias between Fe_OUT(1)_ and Fe_OUT(2)_.

The changes of the magnetic moments of each Fe atoms show clear evidence of such charge transfer. As depicted in Fig. 2b, the Fe atoms that gain electrons lose the net spin moment and the Fe atoms that lose electrons gain the net spin moment in case of FGT monolayers, in which the Fe atoms are near *d*^6^ occupation with S = 1 state. Figure 2c,d show the magnetic moment changes under the electric field for Fe_3_GeTe_2_ and Fe_4_GeTe_2_, respectively. In case of Fe_OUT_ atoms, the changes of magnetic moments show the expected behaviour by the charge transfer. In case of Fe_IN_ atoms, the change of the magnetic moments is governed by ferromagnetic interactions, not by the change of electron occupation numbers as clearly shown in Fig. 2d, where both Fe_IN(1)_ and Fe_IN(2)_ atoms show slight increase of the magnetic moment under the electric field while Fe_IN(1)_ gains the electron and Fe_IN(2)_ loses the electron. It is due to the fact that the charge redistributions are small in Fe_IN_ since they undergo small potential biases.

Figure 2e,f show the changes of magnetic moments of Te atoms under the electric field in Fe_3_GeTe_2_ and Fe_4_GeTe_2_, respectively. The value of each magnetic moment of Te atom changes oppositely to their adjacent Fe atom. This strong antiferromagnetic coupling and hybridization between Fe and adjacent Te implies that the charge redistributions among Fe atoms determine the magnetic properties related to Te atoms through strong Fe-Te hybridizations. Thus, the electric field dependence of MAE can be understood by the Fe atom charge redistribution scenario although the spin–orbit couplings originate from Te atoms.

### MAE change by the electric field

**Figure 3:**
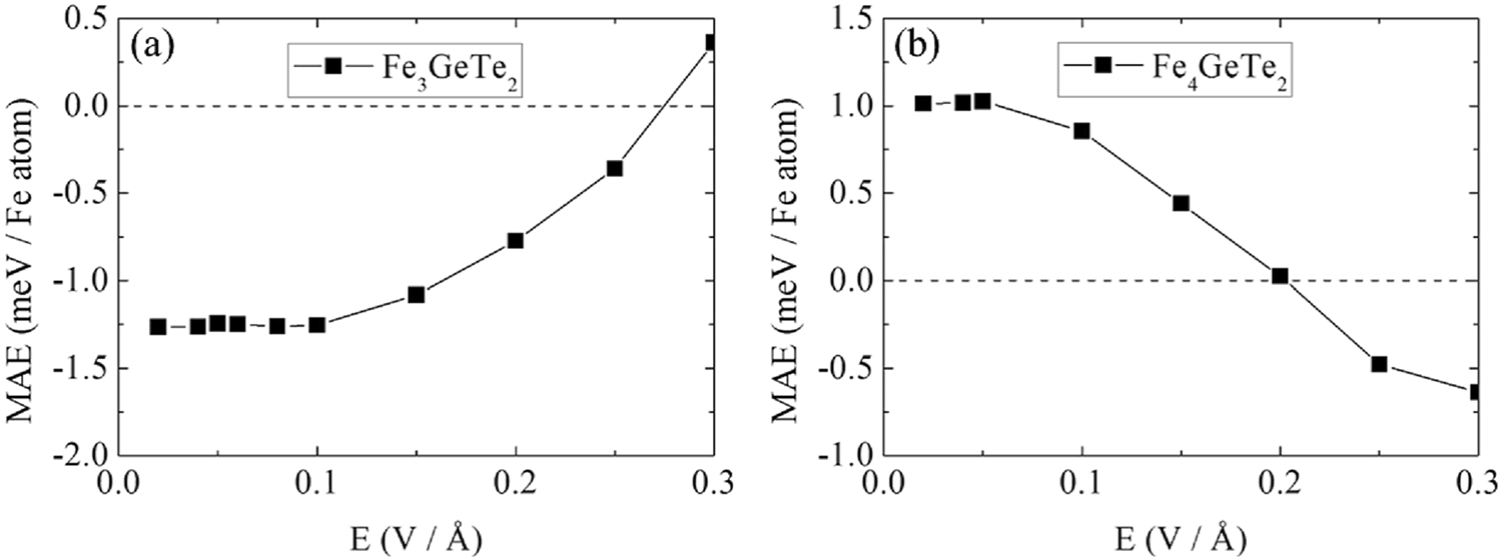
The MAE of (**a**) Fe_3_GeTe_2_ and (**b**) Fe_4_GeTe_2_ defined as E(001)–E(100). The positive (negative) values denote the preferred spin direction along in-plane (out-of-plane) directions.

The changes of the MAE in Fe_3_GeTe_2_ and Fe_4_GeTe_2_ under electric field are shown in Fig. 3a,b, respectively. In case of Fe_3_GeTe_2_, the MAE is about -1.25 meV per Fe atom at zero electric field, indicating uniaxial anisotropy. As the electric field is induced, the MAE increases after plateau of 0.1 V/Å and finally the sign gets changed to positive near 0.25 V/Å, flipping the preferred spin direction from uniaxial to planar. In case of Fe_4_GeTe_2_, the MAE is about 1.0 meV per Fe atom at zero electric field, indicating planar anisotropy. As electric field is induced, the MAE decreases after plateau of 0.05 V/Å and finally sign gets changed to negative near 0.2 V/Å, flipping the preferred spin direction from planar to uniaxial. To summarize, the increase of the perpendicular electric field induces inplane and out-of-plane spin directions in Fe_3_GeTe_2_ and Fe_4_GeTe_2_ respectively. Since Curie temperature is strongly dependent to the MAE in 2-dimensional systems, the stabilization of uniaxial magnetic anisotropy especially in the monolayer or few-layer limit can strongly increase the Curie temperature. Thus, our results show that the induction of perpendicular electric field is expected to increase the Curie temperature of monolayer Fe_4_GeTe_2_ and decrease the Curie temperature of monolayer Fe_3_GeTe_2_.

Since the effect of the electric field in our work is expected to originate directly from the electron redistribution, the MAE change under charge doping should show consistent result to the MAE change under electric field. For the analysis of the MAE change, first, we consider the MAE of the system as a function of the occupancies of Fe atoms, for instance, as ${E}_{MAE}\left({n}_{OUT(1)}, {\mathrm{n}}_{OUT(2)}, {n}_{IN}\right)$ in case of Fe_3_GeTe_2_, where ${n}_{OUT(1)}, {n}_{OUT(2)}$ and ${n}_{IN}$ denotes the electron occupancies of Fe_OUT(1)_, Fe_OUT(2)_ and Fe_IN_, respectively. Secondly, we can consider that the total MAE coupled to each Fe atoms are divisible, so that

1 $${E}_{MAE}\left({n}_{OUT(1)}, {n}_{OUT(2)}, {n}_{IN}\right)={\epsilon }_{OUT}\left({n}_{OUT(1)}\right) + {\epsilon }_{OUT}\left({n}_{OUT(2)}\right) + {\epsilon }_{\mathrm{IN}}\left({n}_{IN}\right),$$

where ${\epsilon }_{OUT}$ and ${\epsilon }_{IN}$ denotes the MAE contributions from Fe_OUT_ and Fe_IN_ atoms, respectively. Here, we assume that the MAE depends only on the charge occupancies of each Fe atoms, based on the previous analysis on Fig. 2. Note that the changes in Te atoms are indirectly covered through the charge occupancies of Fe atoms as discussed in Fig. 2.

Based on the considerations above, the change of the MAE during the charge redistribution among Fe_OUT_ atoms by the electric field can be expressed as

2 $$\Delta {E}_{MAE}={\epsilon }_{OUT}\left(n+\Delta n\right)+{\epsilon }_{OUT}\left(n-\Delta n\right)- 2{\epsilon }_{OUT}\left(n\right),$$

where *n* is the initial occupancy of Fe_OUT_ atoms and $\Delta n$ is the amount of charge transfer from one Fe_OUT_ to the other Fe_OUT_ atom by the electric field. Thus, if ${\epsilon }_{OUT}(n)$ shows convex curve around original occupancy (${\epsilon }_{OUT}(n+\Delta n)+{\epsilon }_{OUT}(n-\Delta n)>2{\epsilon }_{OUT}(n)$), the MAE increases under electric field. Since the contribution to $\Delta {E}_{MAE}$ from ${\epsilon }_{\mathrm{IN}}$ should be much smaller than the contribution from ${\epsilon }_{\mathrm{OUT}}$ due to the reason discussed in the previous analysis on Fig. 2., the convexity of ${\epsilon }_{OUT}(n)$ curve can be considered as equivalent to the convexity of ${E}_{MAE}$ curve.

### MAE change by the charge doping

**Figure 4:**
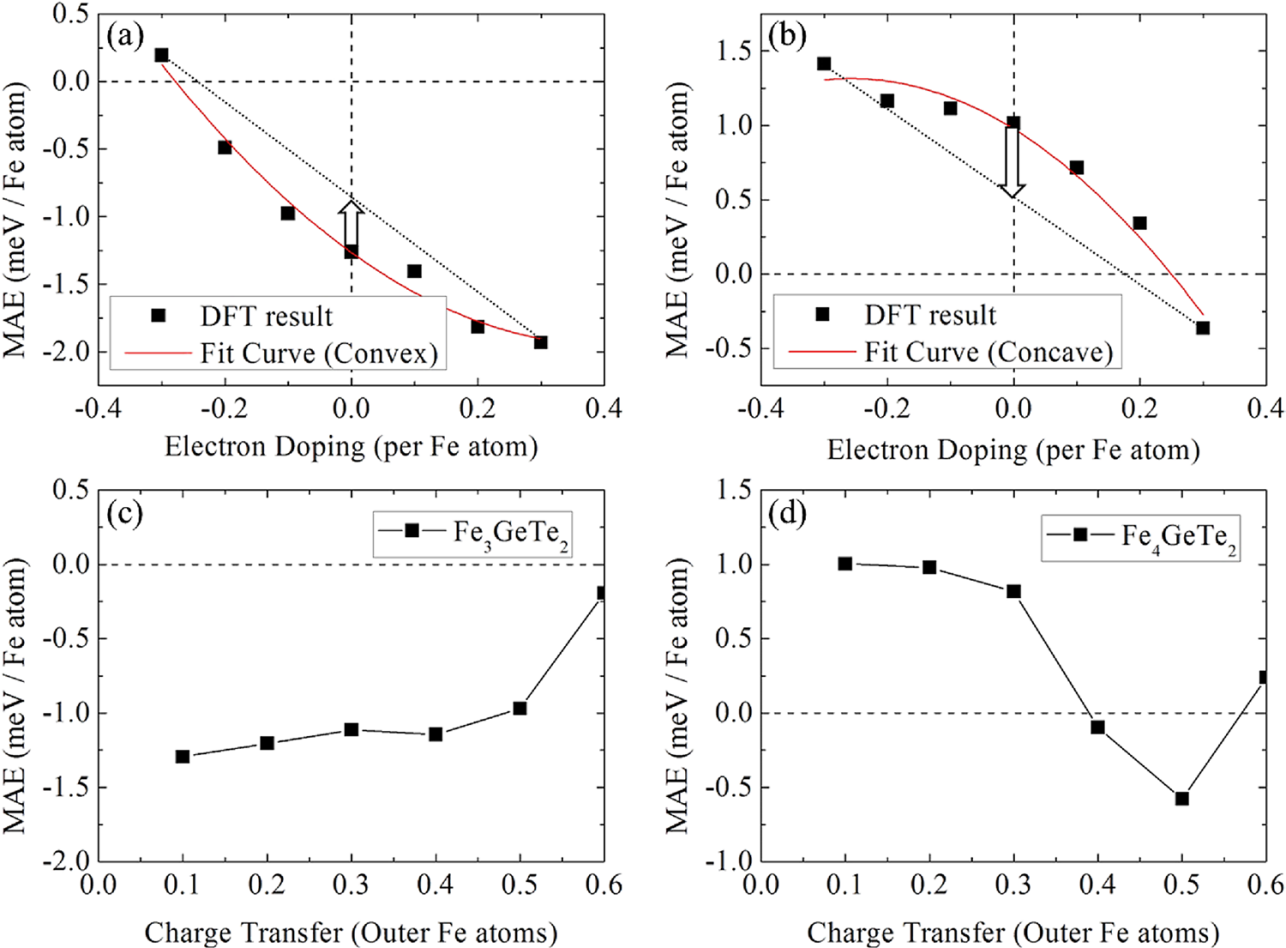
The calculated MAE under charge doping in (**a**) Fe_3_GeTe_2_ and (**b**) Fe_4_GeTe_2_. The red lines denote parabolic fitted curves for DFT results, which shows the convex and concave behaviors of the MAE under charge dopings for Fe_3_GeTe_2_ and Fe_4_GeTe_2_, respectively. The change of the MAE for the asymmetric doping of (**c**) Fe_3_GeTe_2_ and (**d**) Fe_4_GeTe_2_. Fe_OUT(1)_ and Fe_OUT(2)_ atoms are oppositely doped keeping the overall charge constant.

Figure 4a shows the MAE change under charge doping for Fe_3_GeTe_2_. First of all, the result is consistent to the previous work on hole doped bulk Fe_3_GeTe_2_ showing the weakened uniaxial anisotropy as hole doping, although we dealt the monolayers^22^. Furthermore, the convex MAE behavior of Fe_3_GeTe_2_ shows consistency to the increasing MAE under electric field shown in Fig. 3a. Thus, the MAE changes under the charge doping can explain the electric field effect on the MAE. Figure 4b shows the MAE change under charge doping in Fe_4_GeTe_2_. This curve is concave (${\epsilon }_{OUT}(n+\Delta n)+{\epsilon }_{OUT}(n-\Delta n)<2{\epsilon }_{OUT}(n)$), thus the electric field should decrease the MAE of Fe_4_GeTe_2_, which is consistent to the result in Fig. 3b.

**Figure 5:**
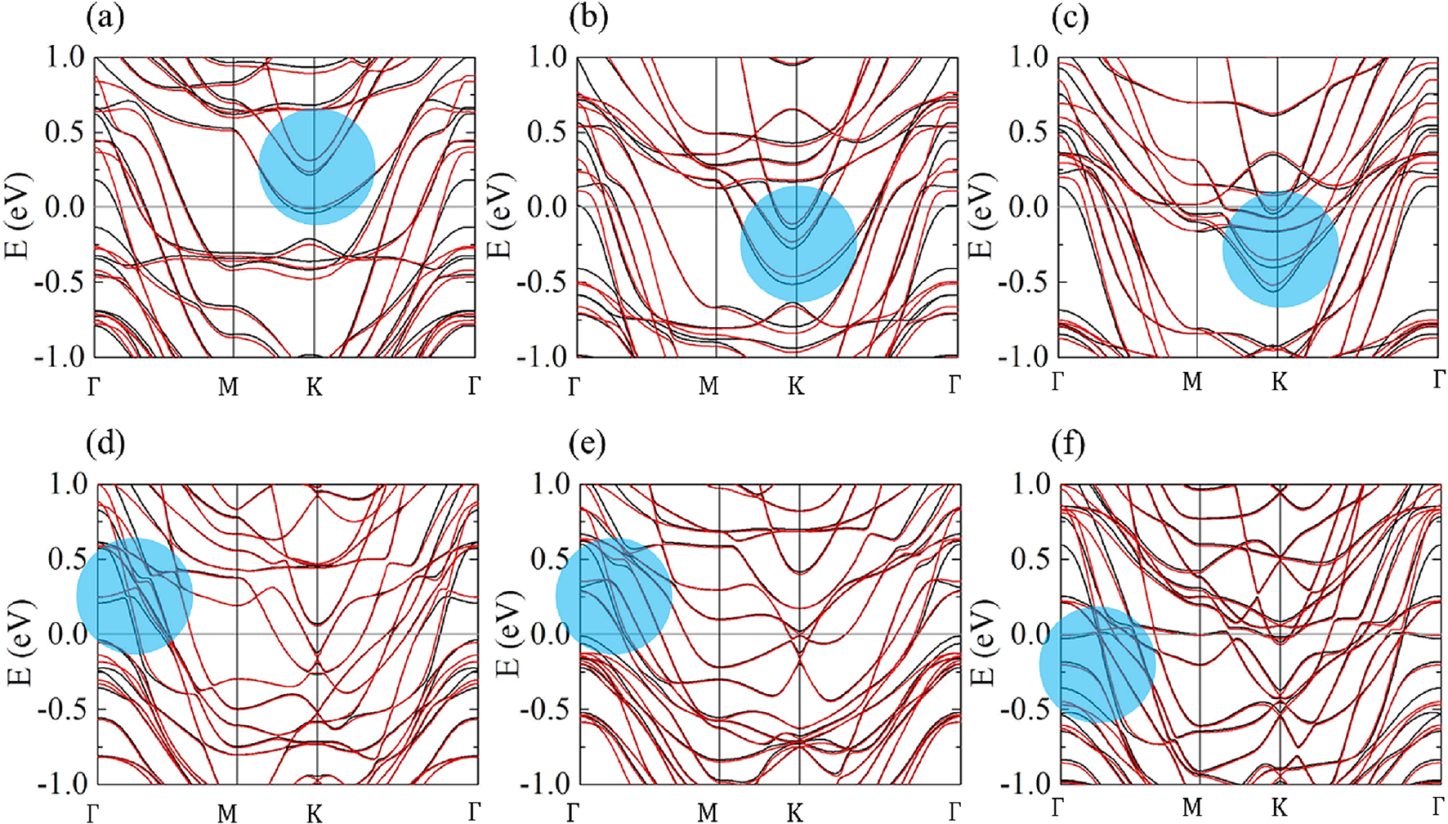
Band structures of FGT with spin direction along 001 denoted by black and 100 denoted by red colour. (**a**–**c**) show the case of Fe_3_GeTe_2_ with doping of − 0.3, 0 and 0.3 electrons per Fe atom, respectively. (**d**–**f**) show the case of Fe_4_GeTe_2_ with doping of − 0.3, 0 and 0.3 electrons per Fe atom, respectively.

Since the key of electric field dependence of the MAE is the convexity of the MAE-doping curve, we also figured out the origin of the convexity to keep track of the MAE changes under the electric field. The different convexity between Fe_3_GeTe_2_ and Fe_4_GeTe_2_ can be analyzed by the shifts of the electronic bands contributing to the MAE as charge doping^22^. Here, the states contributing to MAE can be identified better by the band structures instead of the localized orbitals due to its itinerant characteristics. Figure 5 shows the band structure change under charge doping in Fe_3_GeTe_2_ and Fe_4_GeTe_2_ respectively. In all cases, bands obtained with perpendicular spin direction were denoted as black line and those with planar spin direction were denoted as red line. The bands that the black lines are lower than the red lines contribute to the perpendicular magnetic anisotropy (PMA) and the bands that the black lines are upper than the red lines contribute to the planar magnetic anisotropy. Thus, the change of the occupation of the bands with split black and red lines determine the MAE change by the charge doping^22^. The representative bands contributing to the PMA are denoted as blue regions, which move near the Fermi level by the charge doping.

The same negative sign of the gradient in Fig. 4a,b can be explained by the fact that the bands near the Fermi level mostly to favour the PMA. However the convexity of the curve in Fig. 4a,b were the opposite due to the fact that in Fe_3_GeTe_2_, the blue region is already filled in non-doped case and gets unfilled as hole doping, whereas in Fe_4_GeTe_2_ the blue region is not filled in non-doped case and gets filled as electron doping. Thus in Fe_3_GeTe_2_ hole doping influences stronger than electron doping on MAE and in Fe_4_GeTe_2_ electron doping influences stronger than hole doping.

From these results, we suggest that the convexity of the MAE curve under doping is relevant to the direction of magnetic anisotropy change under electric field. To ensure this idea more certainly, we need to verify that the charge redistributions only in Fe_OUT_ atoms actually affects in the same way. Figure 4c,d show the MAE results of Fe_3_GeTe_2_ and Fe_4_GeTe_2_ respectively, with electrons added in Fe_OUT(1)_ and removed in Fe_OUT(2)_ with same amount by controlling the atomic valence numbers of Fe_OUT(1)_ and Fe_OUT(2)_ individually, mimicking the situation of charge transfer between Fe_OUT_ atoms by the electric field. The results show that the charge redistribution on Fe_OUT_ atoms increase the MAE in Fe_3_GeTe_2_ and decrease the MAE in Fe_4_GeTe_2_ after plateau, being consistent both to the electric field effect and the convexity arguments above. Thus, the idea that the electric field effect on the MAE in these systems is explainable by charge redistributions and that the convexity of the MAE curve under doping determines the direction of the change of the MAE is confirmed.

Since the electron doping is expected to strengthen the uniaxial MAE both in Fe_3_GeTe_2_ and Fe_4_GeTe_2_, Curie temperatures are also expected to increase. To relate this result to experimental situations, substituting Fe to Mn corresponds to hole doping and Fe to Co corresponds to electron doping. Also, the formation of vacancies also can be understood as charge doping. For instance, Te vacancies can effectively dope electrons to Fe atoms and Fe vacancies can effectively dope holes to Fe atoms as discussed in previous study on the MAE change under hole doping^22^. However, Fe vacancies in the bulk FGT systems are expected to influence on Curie temperature through the changes of ferromagnetic exchange or disorder rather than charge doping as pointed out in previous experimental study on Fe_3-x_GeTe_2_^35^.

## Conclusion

The possibility of the electric field control of the MAE of Fe_3_GeTe_2_ and Fe_4_GeTe_2_ monolayers has been investigated by using first principles calculation. Our results showed that the induced electric field along perpendicular direction stabilizes planar magnetic order in case of Fe_3_GeTe_2_ and uniaxial magnetic order in case of Fe_4_GeTe_2_. Moreover, the amounts of the change of MAE were dramatic, allowing the change of easy axis in both systems easily. The origin of the electric field effects on the MAE are well explained through the charge redistribution scheme inside of the layer. While the uniform charge dopings both in Fe_3_GeTe_2_ and Fe_4_GeTe_2_ showed that the electron doping stabilizes the uniaxial magnetic ordering, the charge redistributions among Fe atoms in Fe_3_GeTe_2_ and Fe_4_GeTe_2_ stabilized the planar and uniaxial magnetic ordering, respectively.

Since MAE in two-dimensional systems strongly influence on the Curie temperature, our result also implies that the Curie temperature can be controlled by the electric field in qualitative manner. Quantitative prediction on the Curie temperature change, which is beyond our scope, can be performed by Monte Carlo simulation on anisotropic Heisenberg model^36^.

The MAE control by electric field can be utilized for various practical usages. One certain case is the voltage-transfer torque magnetoresistive random-access memory (VTT-MRAM), suggested for fast and energy efficient memory device for nonvolatile computation without ohmic dissipation, which might support the big-data science and applications in hardware level^37^. Voltage controlled magnetic anisotropy (VCMA) has been studied for thin-film made magnetoresistive tunnel junctions (MTJ) with surface perpendicular magnetic anisotropy (PMA)^37–41^. The value of VCMA coefficient, defined as the MAE change from out-of-plane to in-plane per area divided by the electric field, are about few hundreds ${fJ V}^{-1}{m}^{-1}$ in current status, while it needs to be few ${pJ V}^{-1}{m}^{-1}$ for practical VTT-MRAM application^37^. Our calculations suggest that the VCMA coefficients of Fe_3_GeTe_2_ and Fe_4_GeTe_2_ are about 2 and − 2 ${pJ V}^{-1}{m}^{-1}$ respectively, even in the monolayer with the thickness of nearly 0.5 nm. Alongside with the large absolute value of VCMA coefficient, the FGT materials have one more advantage that it is much free to obtain and manipulate clean layers due to the vdW nature. Although the scale of the electric field in this study is a lot larger than the usual scale of electric field in practical applications, since the same scale of the potential bias is easily reachable with small electric field as the number of the layers increase, the possibility for practical application based on electric field controlled MAE is still open.

These advantageous properties for applications arise from the vdW structure while keeping Fe 3D networks inside of layer. While 3D networks allow intralayer charge redistribution, Te atoms allow large absolute value of VCMA coefficient by offering strong spin–orbit coupling with the hybridization with Fe atoms, working similar to heavy metal doping at the interface. Since the ligand sealed structure is common for the vdW magnetic materials, any vdW materials with 3D metal atom networks are expected to be practically applicable for VTT-MRAM.

Furthermore, various vdW magnetic materials are expected to show drastic MAE change under the electric field in finite multilayers by the mechanism based on the interlayer charge redistribution might work. However, since the other interlayer transitions such as ferromagnetic to antiferromagnetic transitions frequently occur, the study on spin dynamics in such cases might open a new possibility of practical applications.
